# Optimized Birch Bark Extract-Loaded Colloidal Dispersion Using Hydrogenated Phospholipids as Stabilizer

**DOI:** 10.3390/pharmaceutics12090832

**Published:** 2020-08-31

**Authors:** Francis Kamau Mwiiri, Rolf Daniels

**Affiliations:** Department of Pharmaceutical Technology, Eberhard Karls University, Auf der Morgenstelle 8, 72076 Tuebingen, Germany; f.kamau@gmx.de

**Keywords:** colloidal dispersions, birch bark extract, phospholipids, sunflower oil, high-pressure homogenization, interfacial rheology

## Abstract

This study investigated the formulation and processing of aqueous colloidal dispersions containing a birch bark dry extract (TE) as the active substance and hydrogenated phospholipids (Phospholipon 90H) as stabilizer, which can be used in the preparation of electrospun wound dressings. Colloidal dispersions manufactured using a two-stage homogenization process had a bimodal particle size distribution, which was most significantly (*p* < 0.0001) affected by the phospholipid content. The size of the single particles decreased from an average particle size of about 4 µm to a particle size of approximately 400 nm. Dynamic interfacial tension studies performed using a profile analysis tensiometer (PAT) showed that the phospholipids strongly declined the interfacial tension, whereas a further decrease was observed when phospholipids were combined with birch bark extract. Interfacial viscoelasticity properties analyzed using the oscillating drop technique resulted in an increase of both interfacial elasticity and viscosity values. These results indicated that the phospholipids are preferentially located at the lipophilic/water interface and a stable film is formed. Furthermore, the results point to a synergistic interaction between phospholipids and TE. Confocal Raman microscopy (CRM) suggested that the TE is predominantly located in the oil phase and the phospholipids at the interface.

## 1. Introduction

Dry extracts from the outer bark of birch consist mainly of pentacyclic triterpenes, which are known for various pharmacological properties such as anti-inflammatory, anti-viral, anti-cancer activity, and wound-healing effects [1,2,3,4,5,6,7,8]. A well-characterized commercially used triterpene dry extract from the outer bark of birch contains about 80% (*w/w*) betulin and is obtained by accelerated solvent extraction with n-heptane [9]. Other disclosed triterpenes of the dry extract include lupeol (LU), erythrodiol (ER), betulinic acid (BA), and oleanolic acid (OA), as shown in Table 1 [10]. However, the low solubility of these triterpenes in polar and non-polar solvents makes formulation a challenging task, which might limit their therapeutic application [7,11,12]. For topical application, only a few formulation concepts are known. These include cosmetic water-in-oil creams, water-in-oil foams [8], and an oleogel consisting of TE and sunflower oil, which received the European marketing authorization in January 2016 [12]. To the best of our knowledge, an aqueous colloidal dispersion of TE has not been described so far.

However, such a colloidal dispersion would be a prerequisite as we intend to develop a bioactive wound dressing containing colloidal dispersions of birch bark extract through a blend electrospinning technique [13]. In this case, the colloidal TE dispersions will be blended with a suitable water-soluble polymer, e.g., polyvinyl alcohol (PVA) or polyvinyl pyrrolidone (PVP), to create sub-micron-sized electrospun fibers even without further modification of the spinneret.

Phospholipids (PL) are commonly found in all cell membranes and therefore exist in all living organisms where they serve both structural and functional purposes. Lecithin is the most common form of phospholipids, where phosphatidylcholine (PC) is the major component [14,15]. Phospholipids are amphiphilic in nature, having a polar head group and two lipophilic tails. The polar head unit containing a phosphate group can be esterified with an alcohol to an organic molecule such as PC with choline, in phosphatidylethanolamine (PE) with ethanolamine, phosphatidylglycerol (PG) with glycerol, etc. Examples of these phospholipid derivatives can be found here [14]. Due to their amphiphilic characteristics, they have been shown to form bilayers and liposomes or micelles under certain conditions [15]. As a result, PLs are frequently used as emulsifiers in the pharmaceutical, cosmetic, and food industry [14,16,17,18]. Being amphiphilic in nature, they can form a viscoelastic interfacial film that provides excellent colloidal stability [19,20,21,22]. Studies have shown that the dispersion particle size depends on the surfactant concentration whereby the particles significantly decrease in size as the surfactant concentration was increased [23,24,25,26]. Beyond that, many studies suggest that there is a direct interaction between PL and pentacyclic triterpenes [27,28]. This is also supported by preliminary studies in our group indicating that colloidal aqueous dispersions of TE are feasible in the presence of hydrogenated PL. These unique features make phospholipids most suitable to be used in this study in the preparation of colloidal dispersions containing birch bark extract.

Colloidal dispersions, e.g., nanoemulsions or solid-lipid nanoparticles, are widely known as drug carriers for various potential therapeutic applications i.e., oral, ocular, dermal, and parenteral [29,30,31,32,33]. More exclusively, they have been used to incorporate poorly water-soluble drugs, increase their bioavailability, or minimize the side effects of potent drugs [34,35,36,37]. Several methods have been utilized to manufacture such colloidal dispersions. Mostly, they can be formed by first forming a primary coarse dispersion with larger particle sizes in the micron range (1–5 µm) and then after subjecting them to high-energy dispersion methods, e.g., high-pressure homogenization, they can be reduced to the colloidal range (<500 nm). Usually, pre-dispersions formed in the first step by high shear mixing have high variable particle sizes, whereas the subsequent high-pressure homogenization produces dispersions with smaller and also more uniform particle sizes [30,38,39]. However, a simple preparation of TE-based aqueous or oily colloidal suspensions have been reported to be challenging due to the unique structure of the extract particle, which has been described as a “cauliflower-like” structure. These highly porous triterpene particles cannot be crushed by common techniques to reach particles in the colloidal size range, even when high-energy dispersion techniques, e.g., sonication or high-pressure homogenization, were utilized [40].

The aim of this study was (1) to study the role played by PL with respect to the distribution of TE in particular at the oil–water interface, (2) to develop a suitable workflow for the production of aqueous colloidal dispersions containing TE with particle sizes in colloidal range through a homogenization process, and (3) to investigate the influence of PL and sunflower content on particle size reduction.

## 2. Materials and Methods

### 2.1. Materials

Hydrogenated phospholipids from soybean lecithin (Phospholipon 90H) were supplied by Lipoid GmbH (Ludwigshafen, Germany). Sunflower oil was purchased from Caesar & Loretz GmbH (Hilden, Germany). Birch bark extract was obtained from Amryt AG, (Niefern-Öschelbronn, Germany). Reverse osmosis water (ELGA Labwater, Celle, Germany) was used for the preparation of all aqueous solutions.

### 2.2. Experimental Design and Statistical Analysis

All statistical calculations, experimental design generation, and optimization procedures were performed with JMP version 14.2 software (SAS Institute, Cary, NC, USA). A full factorial design of experiment (DoE) was used to generate combinations of formulations with a 3 × 4 design as shown in Table 2. Phospholipon 90H (PL90H) and sunflower oil (SO) content were taken as independent variables, whereas the amount of TE was kept constant at 0.5%. Thereafter, the respective formulations were prepared and assessed for particle size and polydispersity index (PDI). The observed response (particle size (nm)) was analyzed and compared with the predicted values. Beyond that, 3-D surface representations and leverage plots were constructed. The significance of each factor was evaluated by analysis of variance (ANOVA), and P values less than 0.05 were considered significant.

### 2.3. Preparation of Aqueous Colloidal Dispersions

The colloidal dispersions prepared in this work consisted of PL90H, sunflower oil, and TE as the dispersed phase (1–4%) and water as the continuous phase (96–99%). A set of formulations as suggested by the corresponding DoE (Table 2) were prepared with varying concentrations of PL90H (0.5%, 1% and 2.5%) and sunflower oil (0%, 0.3%, 0.5%, and 1%), whereas birch bark extract (TE) concentration was kept constant at 0.5% in all runs. Briefly, during the preparation of each formulation, a pre-dispersion (PD) was prepared by first dispersing PL90Hand TEin water at 70 °C for 30 min under magnetic stirring at 400 rpm using a MR 3001 K magnetic hotplate stirrer (Heidolph, Schwabach, Germany). Then, this mixture was homogenized for 5 min using a rotor-stator system (Ultra Turrax T25, IKA, Staufen, Germany) at 9500 rpm. Thereafter, the formed dispersion was added into the SO and homogenized for 3 min. Finally, the pre-dispersion was homogenized using a high-pressure homogenizer (Emulsiflex C-3, Avestin, Mannheim, Germany) for 8 cycles at a pressure of 100 MPa.

### 2.4. Particle Size Analysis

Dispersion particle size distribution and polydispersity were determined using dynamic light scattering (DLS) (Zetasizer Nano-ZS, Malvern Instruments, Herrenberg, Germany). In general, samples were diluted (1:10,000) with reversed osmosis water in 1.5 mL polystyrene disposable cuvettes prior to analysis to avoid multiple scattering effects. The results are reported as mean particle diameter (Z-average), size distribution by volume, and uniformity of the distribution (polydispersity index (PDI)). All formulations were analyzed on the day of manufacture, and DLS measurements were carried out at 25 °C in triplicate.

### 2.5. Density Measurements

Density was measured at 25 °C by means of a DMA 4500 density meter with an oscillating U-tube (Anton Paar, Filderstadt, Germany).

### 2.6. Interfacial Tension and Elasticity

Dynamic interfacial tension and elasticity was measured using a profile analysis tensiometer PAT-1D (Sinterface Technologies, Berlin, Germany) by applying the buoyant drop method. This method involves imaging the drop and fitting the shape to the Young–Laplace equation. To prepare the test solutions, TE and/or PL90H were dissolved completely in SO by sonication at 65 °C and cooled down to room temperature before performing further experiments. A buoyant drop was formed in a cuvette made of optical glass 35 × 35 × 32 (mm) containing the aqueous phase, and its shape was imaged via a charge coupled device (CCD) camera and fitted to the Young–Laplace equation using the profile drop analysis software provided (Sinterface Technologies, Berlin, Germany). The interfacial tension, given as a function of time, was calculated from the drop shape using the Young–Laplace Equation:

(1) $$\gamma \left(\frac{1}{R_{1}}+\frac{1}{R_{2}}\right)=∆P_{0}+\left(∆\rho \right)gz$$

where $R_{1}$ and $R_{2}$ are main radii of interface curvature, *γ* is the interfacial tension, $∆P_{0}$ is the pressure difference at a reference plane, ∆ρ is the density difference, *g* is the gravitational constant, and *z* is the vertical height measured from the reference plane [41]. Hence, the interfacial tension values in equilibrium were manually extracted from the extrapolation of the measured data by a *γ* (1/√t) plot to infinite time [19]. The diffusion rates of all the samples were also determined from the slope of the plot.

Interfacial rheology was measured by applying the oscillating drop method using the buoyant drop technique with the profile analysis tensiometer PAT-1D (Sinterface Technologies, Berlin, Germany). The principle of the oscillating drop method involves changes of the size of the interface, where PL molecules are located, by continuously altering the drop volume. In a sinusoidal change of drop volume, and thus also the interface, the following can be observed: as the interface narrows, stabilizer molecules desorb from the interface (interfacial tension increases) and during the subsequent enlargement adsorb the molecules again (interfacial tension decreases) [41,42]. Hence, the rheological properties of adsorption layers are expressed by the relationship between the variation of the interfacial tension, from its initial value, and the expansion of the surface area. The interfacial elastic (*E*) and viscosity modulus can be expressed as [43].

(2) $$E=\frac{d\gamma }{d\;\ln(A)}$$

(3) $$\eta_{d}=\frac{d\gamma }{\left(dA/dt\right)/A}$$

where *E* is the interfacial elasticity, *γ* is the interfacial tension, *A* is the area of the interface, and $\eta_{d}$ is the interfacial dilational viscosity.

The experiments were started after 10 min to allow equilibration of the interface. A single frequency of 0.1 Hz was used to study interfacial elasticity and viscosity. A drop volume of 10 µL ± 2 µL was formed at the capillary tip. Subsequently, the data were fitted, and the interfacial elasticity and viscosity values were calculated through a Fourier Transformation (FT) with the associated software. All measurements were performed at 25 °C.

To calculate the interfacial characteristics, the densities of the involved phases are required. The following values have been used: SO (0.91761 g/cm^3^), SO + 0.1% TE (0.91782 g/cm^3^): SO + 0.1% PL90H (0.91782 g/cm^3^), and SO + 0.1% TE + 0.1% PL90H (0.91798 g/cm^3^)).

### 2.7. Confocal Raman Spectral Imaging

A pre-dispersion containing sunflower oil, water, TE, and phospholipids was imaged using an alpha 500R Raman microscope (WiTec GmbH, Ulm, Germany) equipped with a DV401-BV CCD detector, connected via an optical fiber to a UHTS 300 spectrometer and a 532 nm laser excitation source. A 100× air objective (EC Epiplan-Neofluor, Carl Zeiss, Oberkochen, Germany) with a numerical aperture of 0.9 was used to view the sample, while the laser intensity was adjusted to 35 mW. The spectral range covered the fingerprint region between 710 and 1820 cm^−1^ and the region between 2700 and 3580 cm^−1^. The spectra of all single components (PL90H, SO, and TE) were first collected and used as a reference to monitor the presence of each component in the dispersion. Subsequently, the test samples were presented to the microscope on glass slides (VWR-International, Darmstadt, Germany) and covered with a coverslip. Image scans of dispersion using 25 µm × 25 µm area of the surface were taken at an integration time of 0.01 s. Henceforth, color-coded images were obtained by initial cosmic ray removal and spectral background subtraction using the WiTec Project data analysis software 4.1 (WiTec GmbH, Ulm, Germany). By assigning the spectrum of each component to a color, the distribution of all components within the sample can be indicated, resulting in the color-coded image of the scanned area [44].

## 3. Results and Discussion

### 3.1. Confocal Raman Spectral Imaging

**Figure 1:**
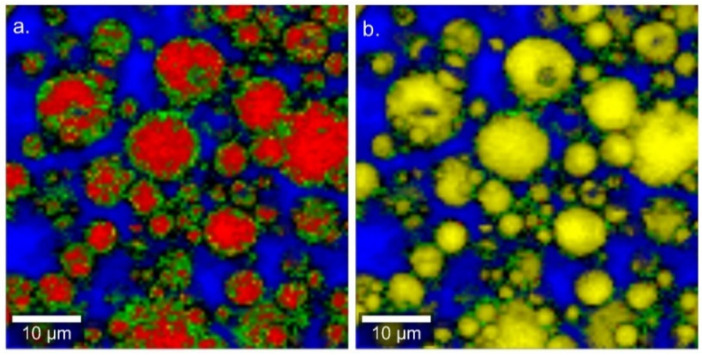
Confocal Raman microscopic color-coded images of aqueous pre-dispersion (**a**) Red: TE, Green: PL90H, Blue: Water, (**b**) shows TE removed in the image to facilitate the better identification of the yellow sunflower oil (SO).

This study focused on colloidal systems consisting of PL, SO, and water, which to the best of our knowledge enabled for the first time the preparation of a colloidal TE dispersion. To get a first insight into the spatial distribution of the components, Raman microspectral imaging was applied to a coarse pre-dispersion consisting of 2.5% PL90H, 1% SO, and 0.5% TE. As can be seen in Figure 1, TE (red) enriches predominantly in the oil phase (yellow), meaning that the extract has high affinity to sunflower oil. On the other hand, no isolated TE particles dispersed in the aqueous phase have ever been detected on Raman images. As expected, the images clearly reveal that the PL90H (green) forms an interfacial layer between the lipophilic droplet (TE and SO) and water (blue). Consequently, this will lead to a reduction of the interfacial tension and stabilizes the dispersion of the two lipophilic components in the aqueous phase. Due to the limited spatial resolution, the Raman microscopic images could neither give hints on a direct interaction of TE and PL90H in these coarse dispersions nor could it seriously be applied to colloidal dispersions.

### 3.2. Interfacial Tension and Viscoelasticity

**Figure 2:**
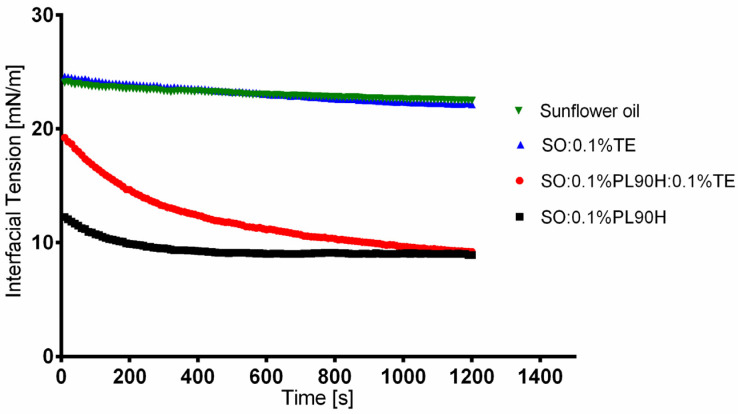
Dynamic interfacial tension curves of PL90H, TE, and PL90H + TE mixture dispersed in SO with water (*n* = 10).

To gain further insights into the potential role of PL90H in the dispersions process and its interaction with TE, we have conducted dynamic interfacial tension experiments at the oil–water interface in the absence and presence of PL90H. Figure 2 illustrates the results of the interfacial tension measurements of SO, reference (21.58 ± 0.27 mN/m), SO + 0.1% TE (19.96 ± 0.39 mN/m): SO + 0.1% PL90H (8.4 ± 0.58 mN/m), and SO + 0.1% TE + 0.1% PL90H (4.75 ± 0.54 mN/m).

It is evident that where PL90H is present, a significant reduction of interfacial tension occurs, whereas TE displays only marginal interfacial activity. From the slope of the plot (interfacial tension versus 1/√t), diffusion rates toward the interface can be calculated. This seems to be of particular interest when comparing the ternary system water, SO, and PL90H with the quaternary system consisting of water, SO, PL90H, and TE. 17.22 mN/m s^−1/2^ or SO + 0.1% PL and SO + 0.1% TE + 0.1% PL90H exhibited the highest diffusion rate of 55.91 mN/m s^−1/2^. This reveals that PL90H and TE strongly interact with each other, probably synergistically forming a film/membrane at the interface, which leads to a faster and more effective reduction of the interfacial tension.

**Figure 3:**
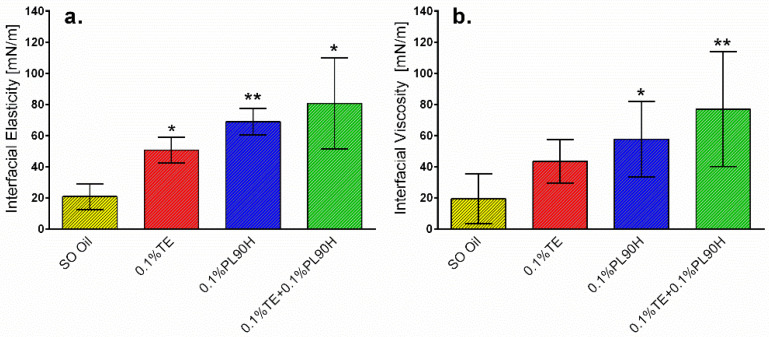
Interfacial elasticity (**a**) and viscosity (**b**) of 0.1% PL90H, 0.1% TE and PL90H + TE dispersed in sunflower oil (Mean ± SD, *n* = 6). statistically significant, * *p* < 0.05; ** *p* < 0.01 vs. sunflower oil (reference).

The interfacial elasticity and viscosity of the test samples obtained from the oscillatory perturbation at a frequency of 0.1 Hz. Pure SO presented low interfacial elastic and viscosity values whereas for samples containing TE and PL90H, both values strongly increased. With an addition of PL90H to triterpene extract, both interfacial elastic and viscosity values significantly shifted toward higher values as compared to reference oil. It seems with the observed higher interfacial elastic values (Figure 3), that PL90H adsorbs at the interface and over the time, a plateau is formed; consequently, the interface becomes more elastic and more structured. Studies have shown that phospholipid molecules can adsorb at the interface, forming a strong elastic film [15,45,46]. Therefore, the obtained high interfacial elasticity values indicate a stable rigid film formation at the interface. Moreover, low elasticity in the interface would require less energy to break the dispersion particles during the emulsification process. High interfacial elasticity is necessary in the stability of dispersions, as a stable film can hinder various instabilities such as the coalescence of dispersion particles from taking place [22,25,47]. Hence, the interfacial elasticity and viscosity can play a key role in the assessment of the deformability of dispersion particles and thus their stability [48]. In this study, the results indicate that a mixture of phospholipid + triterpene extract forms together a combined film with the highest elasticity at the interface, and such a complex again reveals that these two substances interact and do have a synergetic action. Further studies have shown that triterpenes interact with phospholipids (PL) and fluidize lipid membranes controlled by their free polar groups where tetracyclic triterpenes (e.g., cortisol) tend to be located at the head group, whereas pentacyclic ones (e.g., erythrodiol) being more hydrophobic are incorporated deeply in the lipid bilayers [28,49]. Cyclic triterpenes share similar structural properties with cholesterol, and it has been reported that such a monolayer containing cholesterol and PL is highly ordered. On the other hand, binary films of triterpenes and PLs are amorphous [27].

### 3.3. Particle Size Reduction

**Figure 4:**
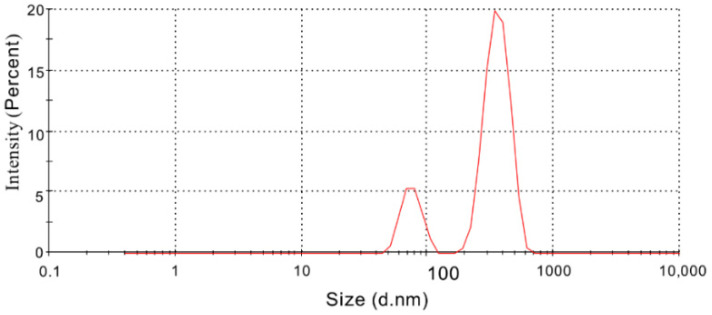
Bimodal particle size distribution by intensity of colloidal dispersion (2.5% PL90H:1% SO:0.5% TE).

The suitability of high-pressure homogenization to produce colloidal dispersions of TE, PL90H, and SO in water was first tested with a formulation consisting of 2.5% PL90H, 0.5% TE, and 1% SO. After mixing and homogenizing with an Ultra Turrax, a mean particle size of 3400 ± 390 nm (pre-dispersion (PD), PDI: 0.9) was achieved. This could be reduced to 400 ± 49 nm (PDI: 0.5) by high-pressure homogenization, yielding a bimodal distribution function. The intensity weighed particle size distribution revealed a major peak at 364 nm comprising 81% and a smaller one at 76 nm representing 19% (Figure 4).

To gain further insight, a DoE has been applied in order to identify the composition variables that influence the particle size distribution of the colloidal dispersions consisting of PL90H, SO, water, and a constant amount of 0.5% TE. The design space has to be restricted with respect to maximal PL90H (<2.5%) and SO concentration (<1%) in order to obtain sufficiently fluid systems necessary for high-pressure homogenization. Table 3 summarizes the respective results.

As expected, and shown in Table 3, pre-dispersions (PD) in all prepared formulations exhibited large particle sizes that were significantly reduced after high-pressure homogenization. Increasing either or both the content of SO and PL90H affected the size and distribution width (expressed as PDI) positively, whereby PL90H had a more pronounced effect. By increasing the concentration of PL90H from 0.5% (1033 nm) to 1% (730 nm) in the system while concentrations of SO and TE are kept constant (0.5%), the particle size could be reduced by a percentage of approximately 30%. A further increase of PL90H to 2.5% revealed a mean particle size of 400 nm which equals to a reduction by ca. 90%.

**Figure 5:**
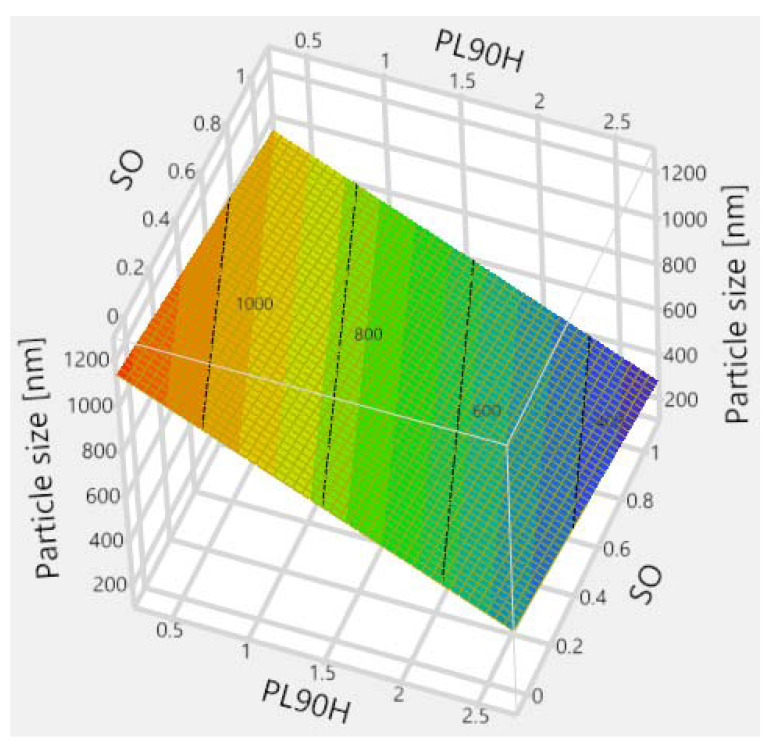
Effect of independent variables on particle size presented in 3D response surface.

**Figure 6:**
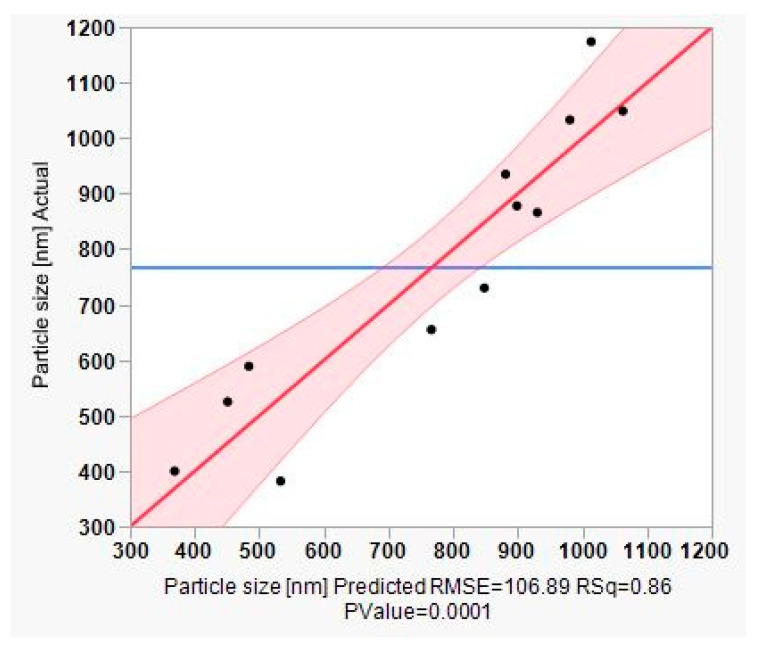
Leverage plot for the whole model of actual vs. predicted particle size.

The whole model actual versus predicted plot with the details of ANOVA (*p*-value < 0.0001, R^2^ of 0.86) in Figure 6 shows that all data points are close to the straight line, indicating that the plot is suitable, and the model fits well. The regression line and the 95% confidence curves crossed the horizontal line at the mean of the response (particle size (nm)). This implies that the whole factorial model with all combined effects (Figure 5) explains a significant proportion of the variation in particle size [50].

**Figure 7:**
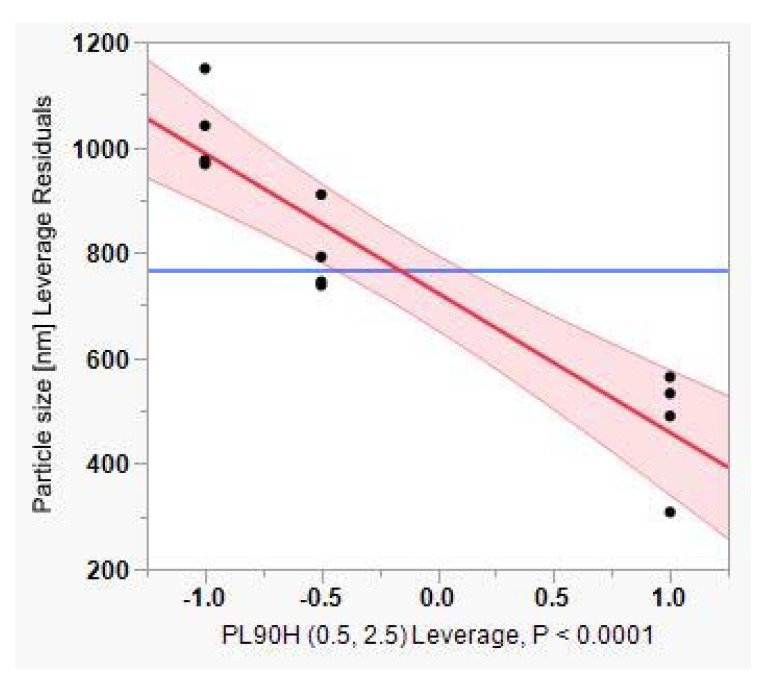
Leverage plot for phospholipon 90H (PL90H).

**Figure 8:**
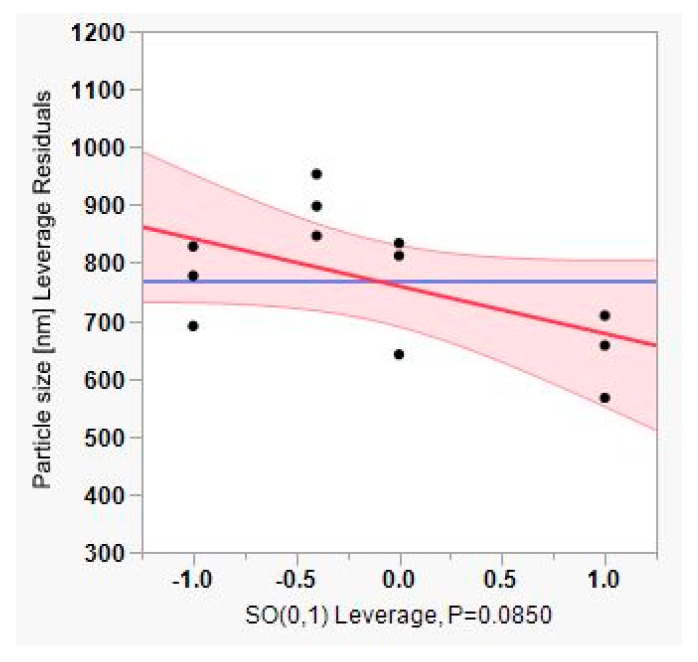
Leverage plot for sunflower oil (SO).

Furthermore, the constructed leverage plots in Figure 6, Figure 7 and Figure 8 revealed that the factor that caused the most significant effect on reducing particle size was the PL90H concentration (*p*-value < 0.0001), followed by the SO (*p*-value < 0.085). In addition, the leverage plot for PL90H showed that both the regression line and the 95% confidence curves crossed the horizontal line; thus, the independent variable significantly influenced and contributed to variations of particle size.

Full model prediction equations for particle size and PDI including independent variables were found to be:

Particle size (nm) = 715.66 − 264.81 × (PL90H − 1.5) − 82.01 × ((SO − 0.5)/0.5)

PDI = 0.63 − 0.13 × (PL90H − 1.5) − 0.025 × ((SO − 0.5)/0.5).

Using the optimization and desirability function of the JMP software, it was predicted that a formulation consisting of 2.5% PL90H, 1% SO, and 0.5%TE allowed to achieve particle sizes as low as 370 nm and with a PDI of 0.5. This is in perfect accordance to our experimental results, where we measured for such an optimal formulation a particle size of 400 nm and a PDI of 0.5.

## 4. Conclusions

A suitable two-stage homogenization process was successfully developed to produce aqueous colloidal dispersions containing TE. The interfacial properties of the PL90H with its SO–TE dispersions were evaluated, and the results showed differences in terms of equilibrium interfacial tension and viscoelastic characteristics, clearly indicating an interaction of PL90H and TE. Confocal Raman spectral imaging allowed visualizing that the two lipophilic components namely, triterpene extract and sunflower oil, were predominantly co-located whereas hydrogenated PL was preferably detected at the oil/water interface. The characteristics of aqueous colloidal dispersions with varied phospholipid-to-sunflower oil ratio were evaluated through particle size measurement. The particle size produced was highly depending on the PL-to-oil ratio. Increasing the PL-to-oil ratio decreased significantly the mean particle sizes. The production of colloidal dispersions with particle sizes of below 1 µm was feasible, as it was our main goal. The optimal composition in the tested design space could be predicted by means of a statistic software.

## Figures and Tables

**Table 1:** Physical–chemical characteristics of the birch bark dry extract (TE) used [8].

TE Composition	Specific Surface Area	Particle Size D50%
Betulin 81.60%, lupeol 2.08%, betulinic acid 3.84%, erythrodiol 1.05%, oleanolic acid 0.97%, betulinic acid methyl ester 0.52%, unidentified substances 9.94%	42 ± 0.4 m^2^/g	5.8 µm

**Table 2:** Design of experiment (DoE) employed for the preparation of aqueous colloidal dispersions.

Batch Code	Pattern	TE (wt %)	PL90H (wt %)	SO (wt %)
**F1**	111	0.5	0.5	0
**F2**	112	0.5	0.5	0.3
**F3**	113	0.5	0.5	0.5
**F4**	114	0.5	0.5	1
**F5**	121	0.5	1	0
**F6**	122	0.5	1	0.3
**F7**	123	0.5	1	0.5
**F8**	124	0.5	1	1
**F9**	131	0.5	2.5	0
**F10**	132	0.5	2.5	0.3
**F11**	133	0.5	2.5	0.5
**F12**	134	0.5	2.5	1

**Table 3:** Observed and predicted particle size and polydispersity index values of aqueous TE dispersions measured using Zetasizer, (Mean ± SD, *n* = 3). PD: pre-dispersion, PDI: polydispersity index.

Batch Code	Pattern	TE (wt %)	PL90H (wt %)	SO (wt %)	PD (nm)	Particle Size [nm]	Dv (d.nm)	Span	PDI	Predicted Particle Size (nm)	Predicted PDI
**F1**	111	0.5	0.5	0	2486 ± 379	1049 ± 176	D10: 93 D50: 510 D90: 769	1.3	0.7	1063	0.8
**F2**	112	0.5	0.5	0.3	1838 ± 405	1174 ± 365	D10: 33 D50: 407 D90: 642	1.5	0.8	1013	0.8
**F3**	113	0.5	0.5	0.5	1738 ± 220	1033 ± 160	D10: 54 D50: 392 D90: 547	1.3	0.8	981	0.8
**F4**	114	0.5	0.5	1	1988 ± 343	878 ± 292	D10: 82 D50: 690 D90: 930	1.2	0.7	898	0.7
**F5**	121	0.5	1	0	1147 ± 167	866 ± 215	D10: 61 D50: 451 D90: 748	1.5	0.7	930	0.7
**F6**	122	0.5	1	0.3	1968 ± 59	935 ± 39	D10: 85 D50: 554 D90: 788	1.3	0.7	881	0.7
**F7**	123	0.5	1	0.5	2982 ± 25	730 ± 114	D10: 36 D50: 72 D90: 403	5.1	0.7	848	0.7
**F8**	124	0.5	1	1	1127 ± 108	655 ± 48	D10: 76 D50: 429 D90: 596	1.2	0.7	766	0.7
**F9**	131	0.5	2.5	0	1799 ± 165	382 ± 35	D10: 58 D50: 299 D90: 554	1.7	0.5	533	0.5
**F10**	132	0.5	2.5	0.3	2420 ± 556	589 ± 36	D10: 415 D50: 678 D90: 1070	0.9	0.4	484	0.5
**F11**	133	0.5	2.5	0.5	1892 ± 253	525 ± 48	D10: 59 D50: 524 D90: 866	1.5	0.5	451	0.5
**F12**	134	0.5	2.5	1	3400 ± 390	400 ± 49	D10: 57 D50: 152 D90: 400	2.3	0.5	370	0.5

Dv: Size distribution by Volume; Span = $\frac{\left(D90-D10\right)}{D50}$.

## References

[1] Aiken C., Chen C.H. (2005). Betulinic acid derivatives as HIV-1 antivirals. Trends Mol. Med..

[2] Haque S., Nawrot D.A., Alakurtti S., Ghemtio L., Yli-Kauhaluoma J., Tammela P. (2014). Screening and Characterisation of Antimicrobial Properties of Semisynthetic Betulin Derivatives. PLoS ONE.

[3] Dehelean C.A., Soica C.M., Toma C.-C., Feflea S., Gruia A.T., Kasa P. (2010). Antitumoral activity of betulin, a compound present in birch tree, in formulations with cyclodextrin. Studia Univ. VgSer. St. Vietii.

[4] Dehelean C.A., Şoica C., Ledeţi I., Aluaş M., Zupko I., Gǎluşcan A., Cinta-Pinzaru S., Munteanu M. (2012). Study of the betulin enriched birch bark extracts effects on human carcinoma cells and ear inflammation. Chem. Cent. J..

[5] Ebeling S., Naumann K., Pollok S., Wardecki T., Vidal-y-Sy S., Nascimento J.M., Boerries M., Schmidt G., Brandner J.M., Merfort I. (2014). From a Traditional Medicinal Plant to a Rational Drug: Understanding the Clinically Proven Wound Healing Efficacy of Birch Bark Extract. PLoS ONE.

[6] Metelmann H.-R., Podmelle F., Waite P.D. (2012). Long-term cosmetic benefit of wound healing by betuline. Am. J. Cosmet. Surg..

[7] Steinbrenner I., Houdek P., Pollok S., Brandner J.M., Daniels R. (2016). Influence of the Oil Phase and Topical Formulation on the Wound Healing Ability of a Birch Bark Dry Extract. PLoS ONE.

[8] Färber A., Daniels R. (2016). Ex vivo Skin Permeation of Betulin from Water-in-Oil Foams. Ski. Pharmacol. Physiol..

[9] Laszczyk M., Jäger S., Simon-Haarhaus B., Scheffler A., Schempp C.M. (2006). Physical, Chemical and Pharmacological Characterization of a New Oleogel-Forming Triterpene Extract from the Outer Bark of Birch (Betulae Cortex). Planta Med..

[10] Krasutsky P.A. (2006). Birch bark research and development. Nat. Prod. Rep..

[11] Jäger S., Laszczyk M.N., Scheffler A. (2008). A preliminary pharmacokinetic study of betulin, the main pentacyclic triterpene from extract of outer bark of birch (Betulae alba cortex). Molecules.

[12] Scheffler A. (2019). The Wound Healing Properties of Betulin from Birch Bark from Bench to Bedside. Planta Med..

[13] Mwiiri F.K., Brandner J.M., Daniels R. (2020). Electrospun Bioactive Wound Dressing Containing Colloidal Dispersions of Birch Bark Dry Extract. Pharmaceutics.

[14] van Hoogevest P., Wendel A. (2014). The use of natural and synthetic phospholipids as pharmaceutical excipients. Eur. J. Lipid Sci. Technol..

[15] Pichot R., Watson R.L., Norton I.T. (2013). Phospholipids at the Interface: Current Trends and Challenges. Int. J. Mol. Sci..

[16] Van Nieuwenhuyzen W. (1981). The industrial uses of special lecithins: A review. J. Am. Oil Chem. Soc..

[17] Hasenhuettl G.L., Hartel R.W. (2008). Food Emulsifiers and Their Applications.

[18] Cabezas D.M., Madoery R., Diehl B.W.K., Tomás M.C. (2012). Emulsifying Properties of Different Modified Sunflower Lecithins. J. Am. Oil Chem. Soc..

[19] Hildebrandt E., Nirschl H., Kok R.J., Leneweit G. (2018). Adsorption of phospholipids at oil/water interfaces during emulsification is controlled by stress relaxation and diffusion. Soft Matter.

[20] Grandell D., Murtomäki L. (1998). Surface Pressure Control of Phospholipid Monolayers at the Water/1,2-Dichloroethane Interface. Langmuir.

[21] Shchipunov Y.A., Schmiedel P. (1996). Phase Behavior of Lecithin at the Oil/Water Interface. Langmuir.

[22] Langevin D. (2000). Influence of interfacial rheology on foam and emulsion properties. Adv. Colloid Interface Sci..

[23] Güell C., Ferrando M., Trentin A., Schroën K. (2017). Apparent Interfacial Tension Effects in Protein Stabilized Emulsions Prepared with Microstructured Systems. Membranes.

[24] Pan L.G., Tomás M.C., Añón M.C. (2004). Oil-in-water emulsions formulated with sunflower lecithins: Vesicle formation and stability. J. Am. Oil Chem. Soc..

[25] Amine C., Dreher J., Helgason T., Tadros T. (2014). Investigation of emulsifying properties and emulsion stability of plant and milk proteins using interfacial tension and interfacial elasticity. Food Hydrocoll..

[26] Henry J.V.L., Fryer P.J., Frith W.J., Norton I.T. (2010). The influence of phospholipids and food proteins on the size and stability of model sub-micron emulsions. Food Hydrocoll..

[27] Broniatowski M., Flasiński M., Wydro P. (2012). Investigation of the interactions of lupane type pentacyclic triterpenes with outer leaflet membrane phospholipids—Langmuir monolayer and synchrotron X-ray scattering study. J. Colloid Interface Sci..

[28] Abboud R., Charcosset C., Greige-Gerges H. (2016). Tetra- and Penta-Cyclic Triterpenes Interaction with Lipid Bilayer Membrane: A Structural Comparative Study. J. Membr. Biol..

[29] Sznitowska M., Janicki S., Dabrowska E., Zurowska-Pryczkowska K. (2001). Submicron emulsions as drug carriers: Studies on destabilization potential of various drugs. Eur. J. Pharm. Sci..

[30] Benita S., Levy M.Y. (1993). Submicron emulsions as colloidal drug carriers for intravenous administration: Comprehensive physicochemical characterization. J. Pharm. Sci..

[31] Fang J.-Y., Leu Y.-L., Chang C.-C., Lin C.-H., Tsai Y.-H. (2004). Lipid Nano/Submicron Emulsions as Vehicles for Topical Flurbiprofen Delivery. Drug Deliv..

[32] Klang S.H., Baszkin A., Benita S. (1996). The stability of piroxicam incorporated in a positively-charged submicron emulsion for ocular administration. Int. J. Pharm..

[33] Aviv H., Friedman D., Bar-Ilan A., Vered M. (1996). Submicron Emulsions as Ocular Drug Delivery Vehicles. Google Patents.

[34] Youenang Piemi M.P., Korner D., Benita S., Jean-Paul M. (1999). Positively and negatively charged submicron emulsions for enhanced topical delivery of antifungal drugs. J. Control. Release.

[35] Rubinstein A., Pathak Y.V., Kleinstern J., Reches A., Benita S. (1991). In Vitro Release and Intestinal Absorption of Physostigmine Salicylate from Submicron Emulsions. J. Pharm. Sci..

[36] Schwarz J.S., Weisspapir M.R., Friedman D.I. (1995). Enhanced Transdermal Delivery of Diazepam by Submicron Emulsion (SME) Creams. Pharm. Res..

[37] Sznitowska M., Dabrowska E.A., Janicki S. (2002). Solubilizing potential of submicron emulsions and aqueous dispersions of lecithin. Int. J. Pharm..

[38] Cortés-Muñoz M., Chevalier-Lucia D., Dumay E. (2009). Characteristics of submicron emulsions prepared by ultra-high pressure homogenisation: Effect of chilled or frozen storage. Food Hydrocoll..

[39] Sjöström B., Bergenståhl B., Kronberg B. (1993). A method for the preparation of submicron particles of sparingly water-soluble drugs by precipitation in oil-in-water emulsions. II: Influence of the emulsifier, the solvent, and the drug substance. J. Pharm. Sci..

[40] Rott C. (2016). Herstellung und Charakterisierung Betulinhaltiger Zubereitungen für Berührungsempfindliche Haut. Ph.D. Thesis.

[41] Miller R., Ferri J.K., Javadi A., Krägel J., Mucic N., Wüstneck R. (2010). Rheology of interfacial layers. Colloid Polym. Sci..

[42] Javadi A., Mucic N., Karbaschi M., Won J., Lotfi M., Dan A., Ulaganathan V., Gochev G., Makievski A., Kovalchuk V. (2013). Characterization methods for liquid interfacial layers. Eur. Phys. J. Spec. Top..

[43] Miller R., Wüstneck R., Krägel J., Kretzschmar G. (1996). Dilational and shear rheology of adsorption layers at liquid interfaces. Colloids Surf. A Physicochem. Eng. Asp..

[44] Heck R., Hermann S., Lunter D.J., Daniels R. (2016). Film-forming formulations containing porous silica for the sustained delivery of actives to the skin. Eur. J. Pharm. Biopharm..

[45] Li J.B., Kretzschmar G., Miller R., Möhwald H. (1999). Viscoelasticity of phospholipid layers at different fluid interfaces. Colloids Surf. A Physicochem. Eng. Asp..

[46] Flasiński M., Hąc-Wydro K., Broniatowski M. (2014). Incorporation of Pentacyclic Triterpenes into Mitochondrial Membrane—Studies on the Interactions in Model 2D Lipid Systems. J. Phys. Chem. B.

[47] Sommerling J.-H., de Matos M.B.C., Hildebrandt E., Dessy A., Kok R.J., Nirschl H., Leneweit G. (2018). Instability Mechanisms of Water-in-Oil Nanoemulsions with Phospholipids: Temporal and Morphological Structures. Langmuir.

[48] Opawale F.O., Burgess D.J. (1998). Influence of Interfacial Rheological Properties of Mixed Emulsifier Films on the Stability of Water-in-Oil-in-Water Emulsions. J. Pharm. Pharmacol..

[49] Tsuchiya H. (2015). Membrane interactions of phytochemicals as their molecular mechanism applicable to the discovery of drug leads from plants. Molecules.

[50] Proust M. (2007). JMP Introductory Guide.

